# Economic and public health impact of decentralized HIV viral load testing: A modelling study in Kenya

**DOI:** 10.1371/journal.pone.0212972

**Published:** 2019-02-27

**Authors:** M. de Necker, J. C. de Beer, M. P. Stander, C. D. Connell, D. Mwai

**Affiliations:** 1 TCD Outcomes Research (Pty) Ltd, Centurion, South Africa; 2 Health Economics Unit, School of Economics, University of Nairobi, Nairobi, Kenya; Burnet Institute, AUSTRALIA

## Abstract

Kenya has the world’s 4^th^ largest HIV burden. Various strategies to control the epidemic have been implemented, including the implementation of viral load (VL) testing to monitor HIV patients on ARVs. Like many resource limited settings, Kenya’s healthcare system faces serious challenges in effectively providing quality health services to its population. Increased investments to strengthen the country’s capacity to diagnose, monitor and treat diseases, particularly HIV and TB, continue to be made but are still inadequate in the face of global health goals like the UNAIDS 90:90:90 which require scaling up of VL tests amid existing constraints. In Kenya, there is an increase in the demand for VL tests amidst these existing constraints. The GeneXpert system is a diagnostic point-of-care technology that can quantify, amongst others, HIV VL. Currently, GeneXpert technology is widely distributed in Kenya for testing of tuberculosis. This study aimed to determine the economic and public health impact of incorporating VL test modules on the existing GeneXpert infrastructure. Markov models were constructed for different populations (non-pregnant adults, pregnant women and children). The scenarios analysed were 100% centralized VL testing compared to 50% GeneXpert plus 50% centralized VL testing, with time horizons of 5 years for the adult and child populations, and 31 months for the pregnant population. Incremental effectiveness was measured in terms of the number of HIV transmissions or opportunistic infections avoided when implementing the GeneXpert scenario compared to a 100% centralized scenario. The model indicated that, for all three populations combined, the GeneXpert scenario resulted in 117 less HIV transmissions and 393 less opportunistic infections. The cost decreased by $21,978,755 for the non-pregnant and pregnant adults and $22,808,533 for non-pregnant adults, pregnant adults and children. The model showed that GeneXpert would cost less and be more effective in terms of total cost per HIV transmission avoided and the total cost per opportunistic infection avoided, except for the pregnant population, when considered separately.

## Introduction

Kenya exhibits one of the worst epidemics of HIV and AIDS in the world [1], with approximately 1.6 million Kenyans living with HIV and approximately 840 000 children orphaned due to the disease [2].

To reduce HIV transmission rates, Kenya had outlined six goals in 2009 that were to be achieved by 2014. The objective of this strategy was to reduce the number of new HIV cases by using evidence-based approaches [3]. There has since been a reduction in HIV mortality [4], but many people are still lost to AIDS [5]. Successful treatment of HIV is dependent on diagnosing patients rapidly, initiating treatment as soon as possible and maintaining adherence to therapies [5].

People living with HIV (PLHIV) should follow the HIV care cascade to fully benefit from antiretroviral therapy (ART). This process begins with HIV testing and ends with monitoring the patients, ensuring that viral suppression has been achieved. PLHIV often do not adhere to the care cascade in the real-world setting [6]. Various methods have been attempted to improve diagnosis, linkage to care, retention in care before initiating treatment, ART adherence and monitoring viral suppression status with the goal of increasing adherence to the HIV cascade [5].

A shortfall is observed in laboratory processing capacity in resource-limited settings. Consequently, many HIV treatment programs are designed in such a way that laboratory monitoring procedures are not prioritized [7]. Focus is instead given to increasing the linkage of PLHIV to care. There is still an ongoing debate on whether resources should be used to increase the initiation of ART in PLHIV rather than improving methods to monitor the effectiveness of and adherence to current treatment regimens [7, 8].

Patients on ART need regular monitoring to assess the effectiveness of treatment and identify emerging drug resistance which, when undetected, results in patients experiencing treatment failure with the consequence of needing more expensive second and third line regimens [9]. This can include immunologic (CD4), clinical (WHO clinical staging) and virological (viral load) monitoring.

Recent World Health Organization (WHO) guidelines suggest that viral load (VL) testing is the preferred way to monitor PLHIV who are treated with ART [8]. Clinical and immunological criteria are poor indicators of virologic treatment failure, and the use of this type of monitoring can result in unnecessary switching of patients to more expensive second line ARTs [9, 10]. Viral load is a more sensitive predictor of treatment failure and is the gold standard for monitoring response to ART [8]. VL testing is not extensively used in resource-limited settings due to factors such as cost, small numbers of laboratories with expertise in measuring VL and difficulty in reliably transporting specimens [11]. In areas where viral load testing is available, it is often costly as the comprehensive cost per viral load test can range from approximately $24.90 to $44.07 (USD) [12].

The GeneXpert system is a point-of-care platform for rapid and simple-to-use nucleic acid amplification tests. The system is a modular, molecular diagnostic platform [13] that has been successfully implemented across the world for the diagnosis of a multitude of infections. This includes Mycobacterium tuberculosis (MTB).

In Africa, GeneXpert is deployed in many countries in both centralized and decentralized health care settings for the diagnosis of MTB and resistance to the TB drug, rifampicin [14]. These capital diagnostic investments have already been made and any other GeneXpert diagnostic test performed using these machines will only incur a variable cost of the test cartridge.

A GeneXpert test has been developed to quantify HIV VL using the same GeneXpert infrastructure.

Given the existing GeneXpert infrastructure investment in Africa and the scalability of the technology (GeneXpert can perform any of the Cepheid molecular diagnostic tests on the same hardware), it is hypothesized that adding HIV VL tests to the existing GeneXpert infrastructure in Kenya could be a cost-effective way of implementing the WHO VL testing guidelines. This was also investigated to assist in effectively scaling up VL testing in line with the WHO 2016 guidelines. Models were constructed to investigate the economic and public health impact of adding VL testing to the existing GeneXpert infrastructure in Kenya.

## Materials and methods

Markov models were constructed in Microsoft Excel with each model corresponding to one of three risk populations, namely non-pregnant adults (15 years and older), pregnant women and children (0 to 14 years). In this cohort model, two scenarios were compared for each of the three population risk pools. The model compared a scenario where 100% of VL testing is performed in centralized laboratories to a scenario where 50% of VL testing is done with GeneXpert plus 50% of VL testing is done in centralized laboratories.

**Fig 1:**
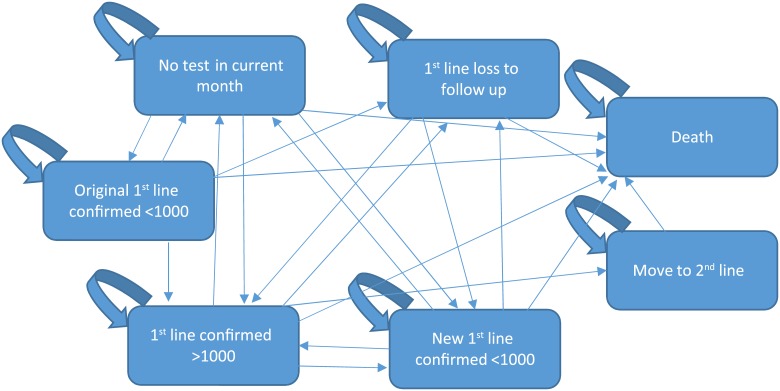
Transition possibilities between main health states.

Six Markov models were constructed to incorporate each scenario for each population risk pool. Patients enter the model at 6 months on antiretroviral treatment (ARVs) (for the adult and child populations) or at 6 months gestation for the pregnant population. The time horizons for the models were 5 years for the adult and child populations, and 31 months for the pregnant population (corresponding to the last 3 months of pregnancy plus 28 months of breastfeeding thereafter). A cycle length of one month was used. Patients flow through the model with different probabilities, through different model states. Fig 1 indicates the main health states used in the model as well as the possible movements between the health states.

At the time of developing the model, the Kenyan local currency to US dollar exchange rate was as follows: 1 Kenyan Shilling equals 0.00979435 US$ [15]. This exchange rate was used in the model.

### Epidemiological inputs

The child and adult populations of Kenya, split between males and females, are shown in Table 1 [1]. The total Kenyan population number was based on the 2017 economic survey [16]. The size of the HIV-positive populations and percentage of HIV-positive patients receiving ARVs are shown in Table 2 [4]. It was assumed that 100% of patients on ARVs would receive a VL test at 6 months. The annual number of new HIV infections in children and adults are shown in Table 3 [4]. These are only considered in the first year of the model, not thereafter. To establish the initial population size, the number of new HIV infections were added to the current HIV-positive population, for each group (adults, pregnant females and children). This was used as the starting population in the Markov models. No new infected people were added to the model during subsequent cycles.

**Table 1:** Child and adult populations of Kenya, split between males and females.

	Total population	Male	Female
**Children (0–14)**	18,555,626	9,306,914	9,248,712
**Adults (15+)**	26,844,374	13,338,681	13,505,692

**Table 2:** Size of HIV-positive population and % HIV-positive and receiving ARVs.

	Size of HIV-positive population	% HIV-positive and receiving ARVs
**Children (0–14)**	98,170	77%
**Non-pregnant adults (15+)**	1,419,537	66%
**Pregnant women**	79,475	75%

**Table 3:** Annual number of new HIV infections in children and adults.

	Annual number of new HIV infections
**Children (0–14)**	6,613
**Adults (15+)**	71,034

The annual number of HIV-related deaths are shown in Table 4 [4]. Additional mortality for patients lost to follow-up was 40% [17].

**Table 4:** Annual number of HIV-related deaths.

	Annual number HIV-related deaths
**Children (0–14)**	5,004
**Non-pregnant adults (15+)**	29,271
**Pregnant women**	1,546

Loss to follow-up for centralized testing was calculated as the average between literature and programmatic data from the Kenya National AIDS and STI (sexually transmitted infections) Control Programme (NASCOP). Literature indicated that the percentage of patients who were 3 or more months late for a scheduled follow-up, but returned within 12 months, was 18.9%. The loss to follow-up rate for patients who were 12 or more months late for a scheduled follow-up, who did not return prior to database closure and were not known to have transferred out or died, was 20.2% [18]. Programmatic data from NASCOP (2013 to 2015) indicated that 12-month retention of patients initiated on ART ranged between 79% to 81% with loss to follow-up rates between 10% and 17%. A final loss to follow-up figure of 27.05% was used in the model base case. This was calculated by adding the 18.9% and the 20.2% mentioned above (equals 39.1%) and then using the average between 39.1% and 15% (NASCOP data).

Based on these figures, 18.9% of patients had the opportunity to return to care at the next testing cycle. According to data for CD4 point-of-care (POC) testing vs centralized testing in Mozambique, loss to follow-up for POC testing was 48% less than for centralized testing [19]. Using this assumption, loss to follow-up for GeneXpert was calculated as 14.1%.

When performing centralized tests, many tests are performed, but the results are not delivered to the patient, due to system errors. This percentage of test performed, where results are not received for centralized monitoring is 46% [19]. This was incorporated as an additional cost, by multiplying the monitoring costs in the centralized arm by 146%. The percentage of test results not received (error rate) for GeneXpert monitoring was 3.1% [20]. This was incorporated as an additional cost, by multiplying the monitoring costs in the GeneXpert arm by 103.1%.

The risk of sexual transmission of HIV, based on the median VL and number of sexual contacts, was calculated based on Wilson et al. [21]. An assumption of 100 sexual contacts per year with a single partner (approximately 8 contacts per month) was used, in line with the assumption used by Estill et al. [22]. For patients with a VL less than 1,000 copies/ml, it was assumed that those patients had a VL of 500 copies/ml [23]. For patients with a VL of more than 1,000 copies/ml, it was assumed that those patients had a VL of 32,000 copies/ml [23]. Children aged 0 to 14 years were assumed to not be sexually active; therefore, HIV sexual transmission for the child population was assumed to be 0.

The frequency of VL testing for virally suppressed and unsuppressed adults and children were every 12 months and every 3 months, respectively. The number of clinic visits per year for stable vs unstable patients are 2 and 6 visits respectively. These were based on Kenyan treatment guidelines [24].

The run time for GeneXpert VL testing is 90 minutes. It was assumed that patients would therefore receive their results on the same day. The turnaround time for centralized VL testing is 15 days [25]. It was assumed that results would only be received in the next month.

The percentage of patients on ARVs with viral suppression (defined as VL <1,000 copies/ml) were 63.2% in the child population [4] and 84.2% in the adult population [26].

Based on the Kenya AIDS Indicator Survey (KAIS 2012) [27] the percentage of adherent patients is 90%, however data on file from a key opinion leader (KOL) on self-reported adherence indicated to use the lower bound of between 80–85%. A value of 80% was used in the base case. The adherence figure was incorporated into the probability of moving to the 2^nd^ line treatment state. The transition matrix calculated the probability of receiving a retest, once a patient is 1^st^ line confirmed, and with a VL above 1 000 copies/ml and multiplied this by the adherence figure for the patients who are not virally supressed. Thus, non-adherent patients will remain on 1^st^ line therapy, and patients would only be switched to 2^nd^ line if they were adherent, but still not virally suppressed.

The number of cases of opportunistic infections (OI) per person-month was calculated by dividing the number of OI cases by the number of person-months [28]. This was split between patients with a VL <1,500 copies/ml (0.003 cases per person-month) and patients with a VL > = 1,500 copies/ml (0.013 cases per person-month), based on the nearest divisions reported in that article.

The mother-to-child transmission rate at delivery was calculated for pregnant women with a VL <10,000 copies/ml (1.4%) and pregnant women with a VL ≥10,000 copies/ml (2.9%) [29]. The mother-to-child transmission rate during breastfeeding (after 6 months, excluding birth) was calculated for mothers with a baseline VL of <10,000 copies/ml (representing suppressed patients), and mothers with a baseline VL of ≥10,000 copies/ml (representing unsuppressed patients) [29]. These rates were subsequently converted to monthly probabilities of transmission (0.1% for VL <10,000 copies/ml versus 0.5% for VL ≥10,000 copies/ml). These classifications were used, as these were the closest VL categories available in that article.

### Cost inputs

The costs of antiretroviral (ARV) unit costs for Kenya was sourced from the National HIV commodity plan for 2016 as the actual prices at which the Kenya Medical Supplies Agency (KEMSA) procures the medicine. For children, patients were assumed to be 3 to 15 years of age, with 50% of children with a weight <35 kg (average 20kg) and 50% of children with a weight ≥35 kg. It was assumed that the cost for efavirenz (EFV) 200 was 1/3 of the cost of EFV 600 as the cost for EFV 200 was not available. The daily cost of 1^st^ and 2^nd^ line ARVs for adults, pregnant women and children in Kenya are shown in Tables 5–8.

**Table 5:** Daily cost of 1st line ARVs for adults and pregnant women.

Combination	Cost per day (USD)
TDF* + 3TC* + EFV* (300 OD + 150 BD + 600 OD)	$0.30
ABC* + 3TC* + EFV* (300 BD + 150 BD + 600 OD)	$0.50
TDF* + 3TC* + ATV/r* (300 OD + 150 BD + 300/100 OD)	$0.66
ABC* + 3TC* + ATV/r* (300 BD + 150 BD + 300/100 OD)	$0.91
AZT* + 3TC* + EFV* (300 BD + 150 BD + 600 OD)	$0.33
AZT* + 3TC* + NVP* (300 BD + 150 BD + 200 BD)	$0.27

* abacavir (ABC); atazanavir/ritonavir (ATV/r); efavirenz (EFV); lamivudine (3TC); nevirapine (NVP); tenofovir disoproxil (TDF); zidovudine (AZT).

**Table 6:** Daily cost of 2^nd^ line ARVs for adults and pregnant women.

Combination	Cost per day (USD)
AZT + 3TC + ATV/r (300 BD + 150 BD + 300/100 OD)	$0.74
TDF + 3TC + ATV/r (300 OD + 150 BD + 300/100 OD)	$0.66
AZT + 3TC + LPV/r (300 BD + 150 BD + 400/100 BD)	$0.91
TDF + 3TC + LPV/r (300 OD + 150 BD + 400/100 BD)	$0.98

**Table 7:** Daily cost of 1st line ARVs for children.

Combination	Cost per day (USD)
ABC + 3TC + EFV (ABC(60)/3TC(30) 3 tablets BD + EFV(200) 1.5 tablets OD)	$0.37
TDF + 3TC + EFV (300 OD + 300 OD + 600 OD)	$0.30

**Table 8:** Daily cost of 2nd line ARVs for children.

Combination	Cost per day (USD)
AZT + 3TC + LPV/r (AZT(60)/3TC(30) 3 tablets BD + LPV/r(80/20) 3ml BD)	$0.84
AZT + 3TC + ATV/r (300 BD + 150 BD + 300/100 OD)	$0.74

The cost of a centralized VL monitoring test ($25) was obtained from KOL opinion and based on internal data presented as a poster at IAS 2017 [30]. The total cost of a GeneXpert test was calculated to be $23.43 by using the percentages from [12]. This was calculated as $14.95 (cost of GeneXpert test, assumed to include, reagents and consumables cost) + $43.42×11.72% (HR cost) + $43.42×0.08% (QC cost) + $43.42×7.74% (cost of relaying results).

The costs of annual laboratory tests were obtained from the Kenya Fees Guidelines for Medical & Dental Practitioners [31] and converted to USD. The average of the minimum and maximum bounds of costs per test were used. The costs are shown in Table 9.

**Table 9:** Cost per test for laboratory tests (in USD).

Laboratory tests	Cost per test–average (USD)
Urinalysis (protein + glucose)	$11.75
Creatinine	$6.46
Glucose	$6.46
Plasma lipid profile	$28.21
**Cost of lab tests done annually**	**$52.89**

The cost of a clinic visit was calculated as the average of clinical and nonclinical labour costs per outpatient visit at a cost of $4.55 [32].

The cost per transmission for patients who become HIV-positive was obtained from literature [33] (cost of newly initiated adult or child ART patient without ARVs) plus the annual cost of 1^st^ line ARVs (for adults or children): $326.88 for adults and $257.41 for children. This is for one year of treatment for a newly diagnosed HIV-positive patient.

The cost of opportunistic infections per patient-year for adults and children were estimated from the CDC and Kenyan Ministry of Health’s ART costing project report [33] as the cost of an established adult ART patient ($120.72), or the cost of an established paediatric ART patient, excluding the cost of ARVs ($123.59).

## Results

Incremental effectiveness was measured in terms of the number of HIV transmissions or opportunistic infections avoided when implementing the GeneXpert scenario compared to a 100% centralized scenario. Incremental costs were calculated as the difference between the GeneXpert scenario and the 100% centralized scenario. Cost-effectiveness was calculated by dividing the incremental cost by the incremental effectiveness.

### Non-pregnant adult population

In the non-pregnant adult population, the GeneXpert scenario resulted in 257 less HIV transmissions and 495 less OIs over 5 years (Table 10). More HIV transmissions were observed for 1^st^ line and 2^nd^ line ARV users due to the fact that in the GeneXpert scenario, more patients remain in care, thus there are more patients being treated with ARVs, thus more patients who can transmit the disease in these states. However, there are less patients who are lost to follow-up, and subsequently transmitting the disease at a higher rate, in the GeneXpert scenario. The reduced number of patients who are lost to follow-up in the GeneXpert scenario outweighs the increased HIV transmissions observed for 1^st^ and 2^nd^ line ARV users. The same applies for OIs where there are more patients on 1^st^ and 2^nd^ line ARV treatment in the GeneXpert scenario, who are at risk of contracting an OI, but less patients who are lost to follow-up. This can be seen in the cost results, where the costs of treating 1^st^ and 2^nd^ line ARV patients are more in the GeneXpert scenario, although monitoring cost is less in the GeneXpert scenario. The GeneXpert scenario resulted in a total cost reduction of $23,752,330 (Table 11). This resulted in a dominant cost-effectiveness result, with the GeneXpert scenario costing less, while being more effective, when based on the incremental cost per HIV transmission avoided and the incremental cost per opportunistic infection avoided.

**Table 10:** Effectiveness of GeneXpert scenario compared to 100% centralized scenario over 5 years in the non-pregnant adult population.

	Centralized (100%)	GeneXpert (50%) + Centralized (50%)	Incremental difference
Number of HIV transmissions—1^st^ line	119 140	119 548	408
Number of HIV transmissions—2^nd^ line	2 001	2 707	706
Number of HIV transmissions—Lost to follow-up	7 280	5 909	-1 371
**Total number of transmissions**	**128 421**	**128 164**	**-257**
Number of OIs—1^st^ line	270 358	271 062	704
Number of OIs—2^nd^ line	4 545	6 150	1 605
Number of OIs–Lost to follow-up	14 884	12 080	-2 803
**Total number of OIs**	**289 787**	**289 292**	**-495**

**Table 11:** Cost of GeneXpert scenario compared to 100% centralized scenario over 5 years in the non-pregnant adult population.

	Centralized (100%)	GeneXpert (50%) + Centralized (50%)	Incremental difference
Cost of treatment—1^st^ line	$125,188,510	$126,937,697	$1,749,187
Cost of treatment—2^nd^ line	$29,859,631	$40,400,704	$10,541,072
VL monitoring cost—1^st^ line	$224,763,876	$188,615,242	-$36,148,634
VL monitoring cost—2^nd^ line	$3,017,945	$3,213,045	$195,100
Cost of HIV transmissions—1^st^ line	$38,944,478	$39,077,725	$133,247
Cost of HIV transmissions—2^nd^ line	$654,011	$884,891	$230,880
Cost of HIV transmissions—lost to follow-up	$2,379,722	$1,931,516	-$448,206
Cost of OIs—1^st^ line	$2,719,801	$2,726,883	$7,082
Cost of OIs—2^nd^ line	$45,726	$61,868	$16,142
Cost of OIs–lost to follow-up	$149,730	$121,529	-$28,201
Total cost (treatment + VL monitoring + OIs + HIV transmissions)	$427,723,430	$403,971,100	-$23,752,330

### Child population

In the child population, the GeneXpert scenario resulted in 46 less opportunistic infections over 5 years (Table 12). More OIs were observed for 2^nd^ line ARV users in the GeneXpert scenario due to the fact that in the GeneXpert scenario, more patients remain in care, thus there are more patients being treated with ARVs, thus more patients who are at risk of contracting an OI, but less patients who are lost to follow-up. The reduced number of patients who are lost to follow-up and the reduction in OIs for patients on 1^st^ line ARVs in the GeneXpert scenario outweighs the increased OIs observed for 2^nd^ line ARV users.

**Table 12:** Effectiveness of GeneXpert scenario compared to 100% centralized scenario over 5 years in the child population.

	Centralized (100%)	GeneXpert (50%) + Centralized (50%)	Incremental difference
Number of OIs—1^st^ line	18 944	18 840	-104
Number of OIs—2^nd^ line	844	1 107	263
Number of OIs–Lost to follow-up	1 068	863	-205
**Total number of OIs**	**20 857**	**20 811**	**-46**

The number of patients who use 1^st^ line ARVs are more in the GeneXpert arm (due to more patients in care), however, the number of patients who are awaiting tests are less, resulting in a decrease in OIs for 1^st^ line patients. This resulted in increased costs for treating 1^st^ and 2^nd^ line ARV patients in the GeneXpert scenario, although monitoring cost is less in the GeneXpert scenario (due to the lower cost of the GeneXpert test). The GeneXpert scenario resulted in a total cost reduction of $829,777 (Table 13). This resulted in a dominant cost-effectiveness result, with the GeneXpert scenario costing less, while being more effective, when based on the incremental cost per opportunistic infection avoided.

**Table 13:** Cost of GeneXpert scenario compared to 100% centralized scenario over 5 years in the child population.

	Centralized (100%)	GeneXpert (50%) + Centralized (50%)	Incremental difference
Cost of treatment—1^st^ line	$7,165,926	$7,185,162	$19,236
Cost of treatment—2^nd^ line	$5,578,621	$7,317,816	$1,739,195
VL monitoring cost—1^st^ line	$15,597,010	$12,988,344	-$2,608,666
VL monitoring cost—2^nd^ line	$560,572	$581,501	$20,929
Cost of OIs—1^st^ line	$195,107	$194,036	-$1,071
Cost of OIs—2^nd^ line	$8,695	$11,406	$2,711
Cost of OIs–lost to follow-up	$11,004	$8,892	-$2,111
Total cost (treatment + VL monitoring + OIs)	$29,116,935	$28,287,158	-$829,777

### Pregnant population

In the pregnant population, the GeneXpert scenario resulted in 76 more HIV transmissions to adults, and 64 more HIV transmissions to babies, over 31 months (Table 14). The GeneXpert scenario resulted in 147 more opportunistic infections (Table 14). There were less patients in care on 1^st^ line ARVs, therefore a decrease in the number of HIV transmissions and OIs in the GeneXpert scenario. However, there were more patients on 2^nd^ line treatment and lost to follow-up, therefore leading to an increase in the number of HIV transmissions and OIs in the total pregnant population or the GeneXpert scenario. The cost increased by $1,773,574 (Table 15). This resulted in a dominated cost-effectiveness scenario, with the GeneXpert scenario costing more and being less effective when based on the incremental cost per HIV transmission avoided and the incremental cost per opportunistic infection avoided.

**Table 14:** Effectiveness of GeneXpert scenario compared to 100% centralized scenario over 31 months in the pregnant population.

	Centralized (100%)	GeneXpert (50%) + Centralized (50%)	Incremental difference
Number of HIV transmissions—1^st^ line	3 486	3 426	-60
Number of HIV transmissions—2^nd^ line	101	203	102
Number of HIV transmissions—Lost to follow-up	466	500	33
**Total number of transmissions (adults)**	**4 053**	**4 129**	**76**
Babies born with HIV—1^st^ line	3 829	3 773	-57
Babies born with HIV—2^nd^ line	94	189	95
Babies born with HIV–Lost to follow-up	431	457	25
**Total number of transmissions (babies transmitted to during birth and/or breastfeeding)**	**4 354**	**4 419**	**64**
Number of OIs—1^st^ line	7 883	7 729	-154
Number of OIs—2^nd^ line	229	462	233
Number of OIs–Lost to follow-up	953	1 022	68
**Total number of OIs**	**9 066**	**9 213**	**147**

**Table 15:** Cost of GeneXpert scenario compared to 100% centralized scenario over 31 months in the pregnant population.

	Centralized (100%)	GeneXpert (50%) + Centralized (50%)	Incremental difference
Cost of treatment	$6,775,561	$9,099,781	$2,324,221
VL monitoring cost	$8,965,966	$8,732,548	-$593,418
Cost of HIV transmissions to adults	$1,324,922	$1,349,758	$24,836
Cost of HIV transmission to babies	$1,120,880	$1,137,337	$16,457
Cost of OIs	$91,200	$92,678	$1,479
Total cost (treatment + VL monitoring + OIs + HIV transmissions)	$18,278,529	$20,052,103	$1,773,574

### Overall results

Combining results obtained for non-pregnant adult, child and pregnant populations, the GeneXpert scenario resulted in 117 less HIV transmissions and 393 less opportunistic infections (Table 16). The cost decreased by $21,978,755 (non-pregnant and pregnant adults) and $22,808,533 (non-pregnant adults, pregnant adults and children) (Table 17). This resulted in dominant cost-effectiveness scenarios, with the GeneXpert scenario costing less and being more effective (Table 18), when based on the total cost per HIV transmission avoided and the total cost per opportunistic infection avoided.

**Table 16:** Effectiveness of GeneXpert scenario compared to 100% centralized scenario over 5 years in the overall population.

	Centralized (100%)	GeneXpert (50%) + Centralized (50%)	Incremental difference
Total number of transmissions	136,829	136,711	-117
Total number of OIs	319,709	319,316	-393

**Table 17:** Cost of GeneXpert scenario compared to 100% centralized scenario over 5 years in the overall population.

	Centralized (100%)	GeneXpert (50%) + Centralized (50%)	Incremental difference
Total incremental cost (adult + pregnant adults)	$446,001,959	$424,023,204	-$21,978,755
Total incremental cost (adults + children + pregnant adults)	$475,118,894	$452,310,361	-$22,808,533

**Table 18:** Cost-effectiveness of GeneXpert scenario compared to 100% centralized scenario for a combination of populations.

Incremental cost per HIV transmission avoided (non-pregnant and pregnant adults)	Dominant
Incremental cost per opportunistic infection avoided (non-pregnant adults, pregnant adults and children)	Dominant

#### Sensitivity analyses

One-way sensitivity analyses were performed where there was uncertainty surrounding certain data inputs (Table 19). Base case inputs were reduced and increased by 5% and 10%. Only the overall results were evaluated in sensitivity analyses. The results are shown in Tables 20 and 21.

**Table 19:** Inputs used in one-way sensitivity analyses.

	Base case	Low	High
Loss to follow-up for centralized testing (+/-5%)	27%	22%	32%
Loss to follow-up for centralized testing (+/-10%)	27%	17%	37%
Percentage of test results not received for centralized monitoring (+/-5%)	46%	41%	51%
Percentage of test results not received for centralized monitoring (+/-10%)	46%	36%	56%
Transmission costs—Adults (15+) (+/-5%)	$327	$311	$343
Transmission costs—Adults (15+) (+/-10%)	$327	$294	$360
Transmission costs—Children (0–14) (+/-5%)	$257	$245	$270
Transmission costs—Children (0–14) (+/-10%)	$257	$232	$283
GeneXpert VL test cost (+/-5%)	$23.43	$22.26	$24.61
GeneXpert VL test cost (+/-10%)	$23.43	$21.09	$25.78
OI costs—Adults (15+) (+/-5%)	$120.72	$114.68	$126.76
OI costs—Adults (15+) (+/-10%)	$120.72	$108.65	$132.79
OI costs—Children (0–14) (+/-5%)	$123.59	$117.41	$129.77
OI costs—Children (0–14) (+/-10%)	$123.59	$111.23	$135.95
Viral suppression rate–Adults (15+) (+/-5%)	84%	79%	89%
Viral suppression rate–Adults (15+) (+/-10%)	84%	74%	94%
Viral suppression rate–Children (0–14) (+/-5%)	63%	58%	68%
Viral suppression rate–Children (0–14) (+/-10%)	63%	53%	73%
Adherence rate (+/-5%)	80%	75%	85%
Adherence rate (+/-10%)	80%	70%	90%
Error rate for GeneXpert testing (+/-5%)	3%	-2%	8%
Error rate for GeneXpert testing (+/-10%)	3%	-7%	13%

**Table 20:** Incremental cost per HIV transmission based on one-way sensitivity analyses.

	Low	High
Loss to follow-up for centralized testing (+/-5%)	-$427,967	Dominant
Loss to follow-up for centralized testing (+/-10%)	-$97,638	Dominant
Percentage of test results not received for centralized monitoring (+/-5%)	Dominant	Dominant
Percentage of test results not received for centralized monitoring (+/-10%)	Dominant	Dominant
Transmission costs—Adults (15+) (+/-5%)	Dominant	Dominant
Transmission costs—Adults (15+) (+/-10%)	Dominant	Dominant
Transmission cost—Children (0–14) (+/-5%)	Dominant	Dominant
Transmission cost—Children (0–14) (+/-10%)	Dominant	Dominant
GeneXpert VL test cost (+/-5%)	Dominant	Dominant
GeneXpert VL test cost (+/-10%)	Dominant	Dominant
OI costs—Adults (15+) (+/-5%)	Dominant	Dominant
OI costs—Adults (15+) (+/-10%)	Dominant	Dominant
OI costs—Children (0–14) (+/-5%)	Dominant	Dominant
OI costs—Children (0–14) (+/-10%)	Dominant	Dominant
Viral suppression rate–Adults (15+) (+/-5%)	-$222,895	Dominant
Viral suppression rate–Adults (15+) (+/-10%)	-$11,698	Dominant
Viral suppression rate–Children (0–14) (+/-5%)	Dominant	Dominant
Viral suppression rate–Children (0–14) (+/-10%)	Dominant	Dominant
Adherence rate (+/-5%)	Dominant	Dominant
Adherence rate (+/-10%)	Dominant	Dominant
Error rate for GeneXpert testing (+/-5%)	Dominant	Dominant
Error rate for GeneXpert testing (+/-10%)	Dominant	Dominant

**Table 21:** Incremental cost per opportunistic infection based on one-way sensitivity analyses.

	Low	High
Loss to follow-up for centralized testing (+/-5%)	Dominant	Dominant
Loss to follow-up for centralized testing (+/-10%)	-$70,512	Dominant
Percentage of test results not received for centralized monitoring (+/-5%)	Dominant	Dominant
Percentage of test results not received for centralized monitoring (+/-10%)	Dominant	Dominant
Transmission costs—Adults (15+) (+/-5%)	Dominant	Dominant
Transmission costs—Adults (15+) (+/-10%)	Dominant	Dominant
Transmission cost—Children (0–14) (+/-5%)	Dominant	Dominant
Transmission cost—Children (0–14) (+/-10%)	Dominant	Dominant
GeneXpert VL test cost (+/-5%)	Dominant	Dominant
GeneXpert VL test cost (+/-10%)	Dominant	Dominant
OI costs—Adults (15+) (+/-5%)	Dominant	Dominant
OI costs—Adults (15+) (+/-10%)	Dominant	Dominant
OI costs—Children (0–14) (+/-5%)	Dominant	Dominant
OI costs—Children (0–14) (+/-10%)	Dominant	Dominant
Viral suppression rate–Adults (15+) (+/-5%)	Dominant	Dominant
Viral suppression rate–Adults (15+) (+/-10%)	-$8,387	Dominant
Viral suppression rate–Children (0–14) (+/-5%)	Dominant	Dominant
Viral suppression rate–Children (0–14) (+/-10%)	Dominant	Dominant
Adherence rate (+/-5%)	Dominant	Dominant
Adherence rate (+/-10%)	Dominant	Dominant
Error rate for GeneXpert testing (+/-5%)	Dominant	Dominant
Error rate for GeneXpert testing (+/-10%)	Dominant	Dominant

For all scenarios investigated in the sensitivity analyses, dominant incremental cost per HIV transmission and per opportunistic infection ratios were obtained (indicating a lower cost and less transmissions or opportunistic infections in the GeneXpert scenario compared to the 100% centralized scenario), except when reducing the loss to follow-up for centralized testing and the viral suppression rate for adults by 5% (for HIV transmissions) or 10% (HIV transmission and opportunistic infections). From the sensitivity analyses it is evident that loss to follow-up for centralized testing and viral suppression rate are sensitive inputs to the model. Reducing the loss to follow-up for centralized testing by 5% and 10% resulted in an increase in the number of transmissions and opportunistic infections in the GeneXpert scenario compared to the 100% centralized scenario. The costs for the GeneXpert scenario, however, remained lower than the costs for the 100% centralized scenario.

The percentage of test results not received for centralized monitoring is a startling 46%. Reducing this figure to 5% results in the GeneXpert scenario being more costly compared to 100% centralised scenario. The incremental cost per transmission and per OI is -R96 015 and -R32 282. This implies that improving the system to reduce the number of tests lost, the use of central laboratories can be cost-effective.

Latest data on the NASCOP dashboard [26] indicate that the proportion of suppressed adults (15+) decreased to 77.56% (accessed on 15 December 2017). This changed the incremental cost per HIV transmission to -$94,185 and the incremental cost per opportunistic infection to -$171,997. This resulted in 99 more HIV transmissions and 55 more opportunistic infections in the GeneXpert scenario, at an increased cost between approximately $9.3 million and $9.45 million. These results indicate that the model is very sensitive to changes in viral suppression rates.

## Discussion

The premise of this study was that, while VL monitoring is the preferred strategy for PLHIV, it is also the most expensive option. Given the sunk cost of capital investment of GeneXpert infrastructure in Africa, we therefore questioned whether the adoption of VL monitoring on the back of this existing infrastructure would be a cost-effective intervention strategy. We answered this question by developing an economic model that mimics the natural history of three risk pools of PLHIV and estimating the economic cost of the intervention on the one hand and the public health impact it achieves on the other hand. To our knowledge, this study is unique in that we could not find any evidence of similar studies that benefit from an existing decentralized POC laboratory infrastructure or that stratifies risk pools into three different cohorts. From a policy perspective, we furthermore believe that the results would be relevant to stakeholders who consider adopting the new WHO guidelines related to monitoring of PLHIV.

In the non-pregnant adult and child populations, introducing decentralized HIV VL testing decreased total and monitoring costs, transmissions and opportunistic infections. ARV treatment costs increased in these populations, pointing towards more patients remaining in care and being retained on 1^st^ and 2^nd^ line ART. Although more people were tested in these populations (76,299 more non-pregnant adults and 1,147 more children tested in the GeneXpert scenario), less VL tests were ultimately performed (1,093,909 less VL tests for non-pregnant adults and 70,770 less VL tests for children in the GeneXpert scenario), due to less tests being lost or not valid in the GeneXpert scenario.

In the pregnant population, 33,936 more women were tested, while 24,800 more VL tests were performed in the GeneXpert scenario. This might be due to the fact that more VL tests are required for pregnant adults (monthly VL monitoring tests) therefore, with increased non-pregnant adults in care, and more VL tests performed, both indicators increased. The benefit gained from POC technology and a reduction in loss to follow-up (LTF), is not that significant in the non-pregnant adults due to frequent testing in the centralized system as well.

A modelling study performed on the Zimbabwean HIV-positive population found that avoidance of the proportion of failed tests or test results not received, differentiated care (by expanding coverage of viral load testing availability) and approaches that increase retention on ART impacts the most on cost-effectiveness of the model [34]. These are features that could manifest with robust point-of-care viral load testing [34]. However, this study also found that a 3-month delay in reporting of results did not have a significant negative impact, which might suggest that the turnaround time for centralized testing observed in Kenya would be acceptable for the patient.

In another economic evaluation of viral load testing in rural Zimbabwe [35], three analyses were performed: simple cost analysis of the initial investment (capital cost), cost-efficiency analysis (cost/output) and a cost -effectiveness analysis (cost/outcomes). The main cost driver from the cost-efficiency analysis was the proprietary lab items (including procurement and storage costs). It was found that GeneXpert VL testing cost $5.16 more than centralized testing per test, based on running costs only. Running costs included the following: sample collection (lab items and transport), cost of running the test (lab items, cost of error, technical staff and other costs including stationary and overheads) and cost of relaying the results. The cost breakdown for centralized testing was as follows: $25.30 ($3.35 for sample collection, $21.91 for running the test and $0.03 for relaying the results). The cost breakdown for GeneXpert VL testing was as follows: $30.46 ($3.30 for sample collection, $27.15 for running the test and $0.00 for relaying the results).

From the cost-effectiveness analysis, the difference in turnaround time for NucliSENS EasyQ (BioMérieux) centralized VL testing vs GeneXpert VL testing was 9 days (10 days for centralized and 1 day for GeneXpert VL testing). In this scenario, GeneXpert VL testing is used specifically for the targeted 27% of ART patients who are in need of a fast turnaround time (pregnant women, adolescents, targeted VL, etc.). Only interlaboratory turnaround time was considered. For GeneXpert VL testing, patients might have to visit clinics again as blood needs to be collected when the samples are sent to the laboratory due to the VL test being performed on plasma instead of dried blood spot (DBS) testing and the preparation and storage requirements for plasma. This was not considered. It is not clear how this would affect turnaround time or loss to follow-up.

In a third Zimbabwean study, using the GeneXpert VL test resulted in shorter overall median turnaround time (1 day compared to 26 days for centralized testing) [36]. An error rate of 3.7% was observed for GeneXpert. ART initiation of patients on enhanced adherence counselling remained high (23 days). The study concluded that health system strengthening is required to complement the use of decentralized systems and ensure the gains obtained in terms of faster turnaround times are also realized further in the health system.

Another study used Activity-Based-Costing methods to compare point-of-care VL testing with centralized VL testing in terms of costs in Kenya [37]. This study obtained average VL unit costs of $29.74 for point-of-care and $24.63 for centralized VL testing. The costs included equipment, human resources, reagents, supplies, training, transportation, quality assurance and recurrent costs. The largest component of unit costs were procurement costs for reagent. Human resource costs were found to be low due to only a small amount of hands-on time needed for workflows at high testing volumes. This study concluded that a cost-effective mix of equipment is required to deliver test results on time, to improve timing of ART assessments and linkages to care. Patient demand for testing, staffing of facilities and the financial capacity to pay for results provided on the same day should guide which VL platform to use.

Comparing results from this analysis against data reported in literature, showed that the annual number of new infections in adults was 71,034 [4], while the number of transmissions to adults from this analysis was 132,474 over a 5-year period (corresponding to 26,495 new infections per year). The transmissions calculated in this model only considers sexual transmission, and not transmission by other means (such as injection drug users, etc) which can contribute to the lower number of infections reported.

For children, the annual number of new infections is 6,613 from literature [4]. In this analysis it was assumed that there is no sexual activity by children in the age group 0 to 14 years, and therefore no transmissions. New infections to children, not included in this model, will be due to babies born from mothers with HIV. From the pregnant model, there was a total of 1,007 babies born with HIV plus 3,347 transmitted to during breastfeeding over the 5-year period (corresponding to approximately 871 new cases of HIV in children in a year).

In addition to the comparisons above, this model is not a dynamic model, therefore no additional patients are added to the model at any cycle, impacting incident cases at every cycle which could also contribute to the difference observed when compared against literature.

## Conclusions

For non-pregnant adults and children, introducing GeneXpert VL testing is a cost-effective way of implementing the WHO guidelines concerning monitoring of treatment failure and can be used to track progress toward the third 90-90-90 target.

In the pregnant model, the number of VL tests performed per annum is every 6 months (twice a year) for suppressed patients and every month (12 times a year) for non-suppressed patients. For the adult and children populations, this input is every 12 months (once a year) for supressed patients or once every 3 months (4 times a year) for non-suppressed patients.

In the pregnant population, due to the turnaround time for centralised testing, all VL tests performed has a 1-month delay until results are received. For non-supressed patients in a centralised model, patients will effectively only receive 6 VL tests per year, where it is determined whether a patient is LTF. In GeneXpert scenario, evaluations to determine LTF occurs every month (no delay in relaying VL test results, thus these patients would return monthly for VL testing). Thus, for the pregnant population, the GeneXpert model has more LTF patients (where there is a higher transmission probability), which implies more HIV transmission from those patients. If the number of VL tests performed in the pregnant model is set equal to that used in the adult and children population, less LTF patients are observed, and overall less transmissions for these patients. The same trends are observed for OIs and transmissions to babies, which, once VL testing frequency is changed, overall GeneXpert will show more benefit compared to centralised model.

In terms of costs, the incremental direct cost (treatment, VL monitoring and OIs) reduces significantly, although the incremental difference is smaller but still positive (more costly).

The time horizon of the pregnant model is 31 months compared to 5 years. The 31 months represents pregnancy and breastfeeding. This cannot be changed as this is not a dynamic model, so once a baby is delivered and the mother is not breastfeeding anymore, she is not removed from the model. This might have an effect on overall costs and effectiveness observed in the pregnant model, compared to that observed in the non-pregnant adult and children populations.

## Supporting information

S1 Table Transition matrix for the GeneXpert adult population. (DOCX)

S2 Table Transition matrix for the centralised adult population. (DOCX)

S3 Table Transition matrix for the GeneXpert pregnant population. (DOCX)

S4 Table Transition matrix for the centralised pregnant population. (DOCX)

S5 Table Transition matrix for the GeneXpert child population. (DOCX)

S6 Table Transition matrix for the centralised child population. (DOCX)
