# Supplementary material for: Economic and public health impact of decentralized HIV viral load testing: A modelling study in Kenya
Source: PLoS One. 2019 Feb 27;14(2):e0212972. doi: 10.1371/journal.pone.0212972 (PMC6392277; doi:10.1371/journal.pone.0212972)
Supplement: S2 Table — (DOCX) [file pone.0212972.s002.docx]

| **Health state** | **No test in current month** | **1st line Lost to follow-up** | **Original 1st line Confirmed <1000** | **New 1st line Confirmed <1000** | **1st line Confirmed >1000** | **Move to 2nd line (adherence incorporated)** | **Death** |
| --- | --- | --- | --- | --- | --- | --- | --- |
| **No test in current month** | 90% | 0% | 4% | 4% | 2% | 0% | 0.16% |
| **1st line Lost to follow-up** | 0% | 80.9% | 0.0% | 15.9% | 3.0% | 0.0% | 0.22% |
| **Original 1st line Confirmed <1000** | 87.4% | 2.7% | 8.2% | 0.0% | 1.5% | 0.0% | 0.16% |
| **New 1st line Confirmed <1000** | 87.4% | 2.7% | 0.0% | 8.2% | 1.5% | 0.0% | 0.16% |
| **1st line Confirmed >1000** | 69.8% | 6.8% | 0.0% | 15.7% | 4.7% | 2.9% | 0.16% |
| **Move to 2nd line (adherence incorporated)** | 0% | 0.0% | 0.0% | 0.0% | 0.0% | 99.8% | 0.16% |
| **Death** | 0% | 0.0% | 0.0% | 0.0% | 0.0% | 0.0% | 100.0% |
